# Co-stimulation of HaCaT keratinization with mechanical stress and air-exposure using a novel 3D culture device

**DOI:** 10.1038/srep33889

**Published:** 2016-09-27

**Authors:** Moon Hee Jung, Sang-Myung Jung, Hwa Sung Shin

**Affiliations:** 1 Department of Biological Engineering, Inha University, Incheon, 402-751, Korea

## Abstract

Artificial skin or skin equivalents have been used for clinical purpose to skin graft and as substitutes for animal experiments. The culture of cell lines such as HaCaT has the potential to produce large amounts of artificial skin at a low cost. However, there is a limit to keratinization due to the restriction of differentiation in HaCaT. In this study, a culture device that mimics the *in vivo* keratinization mechanism, co-stimulated by air-exposure and mechanical stimulation, was developed to construct skin equivalents. The device can reconstruct the epidermal morphology, including the cornified layer, similar to its formation *in vivo*. Under the condition, epidermis was differentiated in the spinous and granular layers. Formation of the stratum corneum is consistent with the mRNA and protein expressions of differentiation markers. The device is the first of its kind to combine air-exposure with mechanical stress to co-stimulate keratinization, which can facilitate the economically viable production of HaCaT-based artificial skin substitutes.

Skin is the largest organ in the outer covering its body. When damaged by diseases, wounds, or burns, the skin must be regenerated to protect the body from bacterial infections. Artificial skin substitutes used in wound dressings or transplantations help damaged skin to heal rapidly. Furthermore, the increasing ethical concerns associated with animal tests in the cosmetics and pharmaceutical industries have made it necessary to develop skin alternatives as a substitute for animal testing^1^.

An important part of artificial skin production is the process of formation of the stratum corneum, which is the layer that protects skin from external agents. The stratum corneum is composed of keratinocytes, which is one of the epidermis layers. Cell division repeatedly occurs in the basal layer and stops in the spinous layer. When the cell nucleus disappears in the granular layer that contains keratohyalin granules, a cornified layer is formed. The stratum corneum, which is composed of dead cells, is continually removed when a new layer is generated from underneath^2^,^3^. When reconstructing skin artificially, it is important to develop methods for mass production with low costs that are capable of keratinization through terminal differentiation. Several methods have been studied that induce differentiation of keratinocytes to reproduce the stratum corneum, including adjusting calcium and serum concentrations of culture media^4^,^5^ or dynamic stimulations through suspension culture^6^,^7^. The use of primary human keratinocytes is limited by their short lifespan and high cost of production^8^,^9^. As a result, the self-renewable keratinocyte cell line, HaCaT, has been extensively investigated for its ability to undergo differentiation through a variety of methods that can induce apoptosis, including ceramide preprocessing or fibroblast co-culture^10^,^11^. However, owing to their low differentiation potential compared to primary keratinocytes, HaCaT cannot be easily used to regenerate the stratum corneum^9^.

Air-exposure and mechanical stress are the typical methods for cornification of skin equivalents or keratinocyte differentiation. When HaCaT cells are exposed to air to induce stratification of the epidermis, they express markers such as keratin, filaggrin, and loricrin, which are the major proteins in the stratum corneum of the skin^12^. Although epidermal tissue formation is possible using HaCaT three-dimensional (3D) culture^13^, HaCaT cells produced by 3D culture are insufficient for stratum corneum formation relative to engineered-constructs of primary human epidermal keratinocytes. Specifically, it was difficult to observe whether it had reproduced the full layers of skin relative to those observed *in vivo*.

Human skin is exposed to various mechanical stimuli that induce mechanosensitive signals^14^, which cause changes in self-renewal or differentiation^15^. Subjecting keratinocytes to mechanical stress has been found to induce changes in proliferation^16^,^17^ or differentiation^18^. Cytosolic influx of Ca^2+^ can occur via membrane distortion of HaCaT, followed by ATP release, which induces production of the stratum corneum by promoting terminal differentiation of HaCaT^19^. Thus, studies have investigated the differentiation of HaCaT via various types of mechanical stress. However, these studies did not utilize 3D skin culture to stimulate mechanical stress. In mechanical stimuli studies, most cells were cultured in monolayer, after which changes in the function and morphology of cells were investigated^16^,^18^. Hence, it is necessary to develop a 3D culture system capable of inducing mechanotransduction *in vitro*.

This study was conducted to develop a method for artificially constructing the complete stratum corneum till the terminal differentiation stage using HaCaT for 3D skin culture. To accomplish this, we developed a novel device for providing a mechanical stimulus that can induce differentiation of HaCaT in 3D, in addition to providing air exposure.

## Results

### Schematic design of the device that supports mechanical stress and provides air-lift to keratinocytes

**Figure 1:**
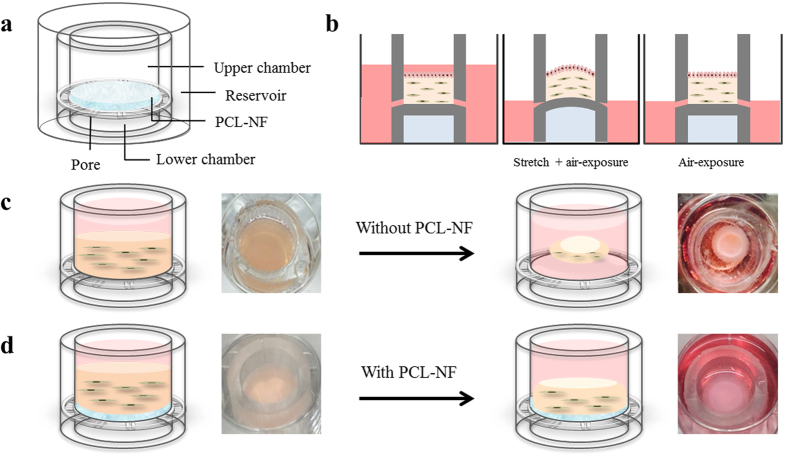
Schematic design of 3D culture device providing mechanical stress and air-exposure to cells. (**a**) An all-in one device was fabricated using PDMS. The device fits into 6-well plates and has 11 holes around the wall to diffuse media between reservoir and upper chamber. (**b**) Cultivation process of 3D skin under mechanical stretch and air-exposure. For differentiating HaCaT, culture medium was removed out of reservoir. The 3D skin was cultured with intermittent stretch for 14 days by injecting water in the lower chamber. (**c**) Collagen matrix without nanofiber was shrunk in the media in 24 h. (**d**) Collagen matrix with nanofiber was fixed by nanofiber in place after 24 h.

We developed a device that induces differentiation of keratinocytes by applying air-exposure and mechanical stimulation simultaneously, to regenerate an artificial test skin model that could be used as a substitute for animal experiments. Figure 1 shows a schematic diagram of the device. Media flows between reservoir and the upper chamber through 11 pores beside the membrane to provide nutrients to cells or air-exposure (Fig. 1a). By adjusting the amount of media in the reservoir, HaCaT is exposed to the air for keratinocyte differentiation. The membrane of the device is expanded by injecting water into the lower chamber for cellular mechanotransduction. As the material of device is made of a flexible PDMS polymer, the expanded membrane blocks inflow of media by closing off the holes. Due to this feature of the device, the media level for air-exposure can be easily maintained and it can provide controlled air-exposure under mechanical stretch (Fig. 1b, Supplementary Video S1). This study is the first trial to demonstrate whether co-stimulation of air-exposure and radial strain is effective for keratinization of HaCaT. For this work, radial strain, defined as equation (1), was adjusted to 15% in the range of 10 to 20%, since excessive strain was known to invoke cell death^20^,^21^,^22^ (Supplementary Fig. S1). Intermittent stretches were cyclically applied for 1 min every 30 min until cell harvesting.

Collagen was used as a scaffold for reconstructing 3D skin. However, collagen scaffold has a limitation because collagen-cell mixture can get readily deformed by reciprocal interaction between collagen and the mixed cells; cell proliferation is also inhibited by the collagen deformation^23^. Therefore, polycaprolactone nanofiber (PCL-NF) was applied to the collagen matrix for stabilizing the scaffold for the fibroblast culture (Fig. 1c,d, Supplementary Fig. S2). As seen in Fig. 1c, once mixed with fibroblasts on the PDMS membrane, free collagen gradually contracts within 1 day. However, PCL-NF applied to collagen secured its structure and kept its morphology intact 1 day after collagen-fibroblast mixture was smeared onto the pre-fixed NF (Fig. 1d).

### Combinatorial stimulation of mechanical stress and air-exposure improved formation of cornified layer of differentiated HaCaT

**Figure 2:**
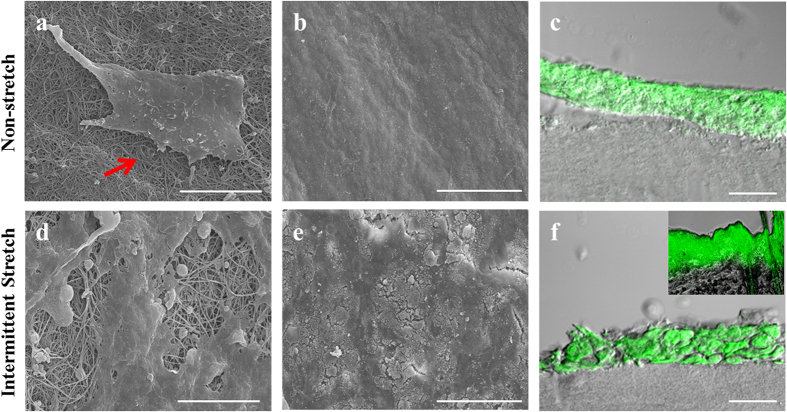
Morphological comparison of the regenerated epidermis under co-stimulation with mechanical stress and air-exposure. SEM images showing epidermis morphology of 3D skin cultured under non-stretch or intermittent-stretch conditions. (**a**,**b**) Collagen fiber (arrow) was covered with smooth epidermis of HaCaT under air-exposure only condition. (**d**,**e**) Collagen fiber was covered with tough stratum corneum of HaCaT differentiated under co-stimulation with intermittent stretch and air-exposure. (**c**,**f**) Epidermal layer was labeled using FITC-conjugated nanoparticles. (**c**) Nanoparticles were deposited into the epidermal layer but distinguishable granular layer was not detected. (**f**) Spinous or granular layers were confirmed, stained with FITC-labeled nanoparticles. Inside figure is the rat skin which was deposited with FITC-labeled nanoparticles. But, Nanoparticles also were not penetrated to the dermal layer of rat skin. (Bar = 10 μm (**a,d**), 100 μm (**b,e**) and 50 μm (**c,f**)).

The top surface of the reconstructed skin was observed by SEM to confirm the formation of the stratum corneum. Collagen matrix revealed a fibrous structure after gelation (Fig. 2a,d). A tougher stratum corneum with higher dimension was observed in intermittent stretch condition (IS) (Fig. 2d,e). To confirm if the dermal layer of the fibroblast is sufficiently concentrated to resist permeation of nanoparticles, it was topically treated with fluorescein isothiocyanate (FITC)-labeled nanoparticles. The chitosan coated-nanoparticles containing ceramide were around 186 nm (data not shown). As a result, fluorescence was detected only in the epidermal layer of HaCaT culture, but not in the dermal layer (Fig. 2c,f), which implied that the reconstructed dermal layer was highly concentrated due to which nanoparticles are unable to or rarely permeate it similar to the *in vivo* dermal layer (inside figure, Fig. 2f). In the epidermal layer under IS, the fluorescent image showed the distinguishable morphology of spinous or granular layers. This result implied that HaCaT was keratinized successfully. In contrast, cells under non-stretch condition (NS) did not reveal the characteristic and distinguishable layers.

### Comparative analysis of keratinocyte differentiation in view of transcript-level studies

**Figure 3:**
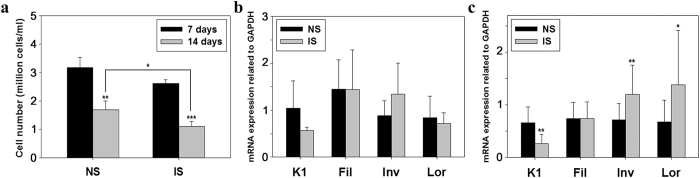
Confluency and qRT-PCR analysis of keratinized 3D skins. Nucleated cell number was compared between NS and IS (**a**). mRNA levels of differentiation markers were quantified at 7 days (**b**) and 14 days (**c**). (**a**) Nucleated cell number on day 14 in IS decreased than NS. (**b**) Differentiation markers did not show significant differences between NS and IS. (**c**) Expression of K1 was lower but those of Inv and Lor were higher in IS. These findings indicate that cells were keratinized following transition process from basal layer to granular layer. Further, the increased Lor implies that HaCaT reached terminal differentiation. Fil did not show significant differences (*p < 0.05, **p < 0.01, ***p < 0.001).

Cell counting and qRT-PCR were conducted to confirm the confluency and keratinization of cultured tissues. As seen in Fig. 3a, number of nucleated cells under NS and IS decreased to 53% and 43% from 7 days to 14 days, respectively (P-values were 0.0052 and 0.0002, respectively). Compared with NS, IS was significantly less nucleated cell number at day 14 (P value 0.048). In the case of qRT-PCR, expression levels of differentiation markers on day 7 did not show significant differences between NS and IS (Fig. 3b). Whereas, on day 14, IS increased the expressions of involucrin and loricrin by 1.7 and 2 fold respectively, relative to NS (P-values were 0.0031 and 0.011, respectively). Expression of keratin 1 decreased (P-value 0.003) and filaggrin expression remained unchanged (Fig. 3c).

### Comparative analysis of keratinocyte differentiation in view of protein-level and lipid-level studies

**Figure 4:**
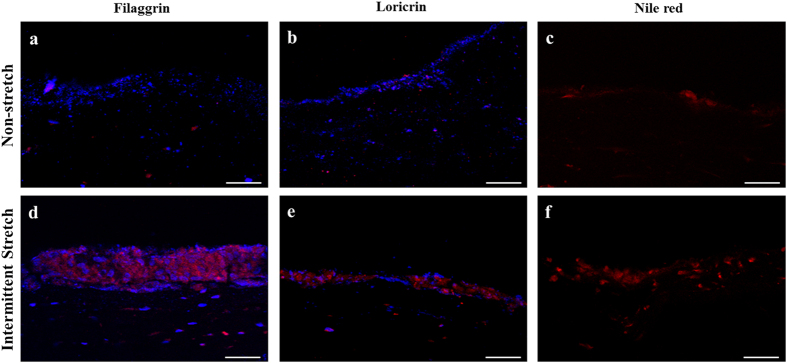
Comparison of expressions of differentiation markers and formation of cornified envelops of 14-day cultured 3D skins between non-stretch and intermittent stretch conditions. Filaggrin (**a**,**d**) and loricrin (**b**,**e**) were immunostained in red and the nucleus was stained in blue. Cornified envelops (**c**,**f**) were identified by Nile red staining of lipids. (**a**,**b**) Both filaggrin and loricrin were less expressed under air-exposure without stretch. (**d**,**e**) Both filaggrin and loricrin were highly expressed under air-exposure with intermittent stretch. (**c**) Cornified envelops were rarely detected under air-exposure only condition. (**f**) High density of cornified envelops was detected with high lipid contents under co-stimulation with intermittent stretch and air-exposure. Bar = 50 μm.

Histology of cultured 3D skins over 3 weeks was analyzed by immunocytochemistry and Nile red staining (Fig. 4) and fluorescent intensities were quantified (Supplementary Fig. S3). 3D skins were successfully reconstructed on the culture device, regardless of the applied mechanical stimulation. The thickness of the skin was 200–300 μm and epidermal layer was around 50 μm in both IS and NS. An immunocytochemistry assay was then conducted to confirm the expressions of filaggrin and loricrin as differentiation markers. Expressions of filaggrin and loricrin were higher under IS (Fig. 4a,b,d,e, respectively). These results were in accordance with those of Nile red staining, identifying lipid contents of the stratum corneum. Strained 3D skin on the device showed high lipid contents and a denser epidermis than that of NS (Fig. 4c,f). After quantifying fluorescence intensities of ICC and Nile red, filaggrin, loricrin, and lipids were confirmed to be increased by 5.6, 6.0, and 2.9-fold in IS than NS, respectively (P-values were 0.0001, 0.0001, 0.0035, respectively) (Supplementary Fig. S3).

## Discussion

Artificial skin substitutes were reconstructed using a novel culture system that provides both mechanical stress and air exposure. Primary keratinocytes have well been established as 3D skin substitutes, but its mass production is cost-prohibitive. Although culture methods using the keratinocyte cell line, HaCaT, have been attempted, no successful methodologies have been reported to date.

The device developed in this study allowed air exposure by adjusting the media volume of the upper chamber. Furthermore, when pressure was applied to the lower chamber through pump-driven flow, membrane expansion occurred, resulting in mechanical stress on the cells. In order to control the pressure level and duration of applied stress to the culture device, the pumping strategy to the lower chamber was automated using Arduino-assisted pump, and the pressure was equally distributed to 6 devices, which were subjected to identical stimulus. Another important step in the fabrication of the device was to construct a 3D scaffold matrix for fibroblast culture on the membrane. Unlike free collagen scaffold, nanofiber-assisted collagen matrix secured a bigger dimension by preventing collagen contraction. The elastic PCL nanofiber can be regarded as a building block which resists collagen contraction. In addition, C-phycocyanin was incorporated into the PCL-NF as it has already proven to provide beneficial effects on long-term culture of fibroblasts in our previous study^24^.

Keratinization is a transformation process of keratinocytes from the basal layer to spinous and granular layers, finally reaching the stratum corneum incorporated with lipid and keratinized proteins. Inferences drawn from the spinous and granular layers under IS, indicated that combinatorial stimulation of intermittent stretch and air-exposure could be more effective in regenerating *in vivo*-like epidermal layer. On day 7, confluency of cells was not significantly different between NS and IS, which correlated with the fact that mRNA levels of differentiation markers were not significantly different. However, the declined number of nucleated cells on day 14 in IS implies that a cellular transition dramatically occurred during epidermis formation in HaCaT under IS. This assertion is supported by variations of differentiation markers; the cultured 3D skin under IS revealed enhanced expressions of involucrin and loricrin and reduced expressions of keratin 1. When cells are transformed from spinous cells to granular cells, keratin 1 in the supra basal layer decreases and differentiation factors such as involucrin increase^25^,^26^. Moreover, increasing loricrin level reveals that more terminal differentiation occurred under the stressed environment. The apoptosis process for keratinization progresses from the granular layer. During this process, differentiation markers like filaggrin become upregulated, which involves variations of cellular morphology and nuclear integrity, finally resulting in the disruption of cytoskeletal filaments^27^,^28^. In addition, strained tissue showed more cornified layer comprising higher levels of lipids than non-stretched tissue (Fig. 4f, Supplementary Fig. S3). When the spinous layer is formed and then transformed to granular layer, lipids are synthesized and form covalent bonds with the cornified-envelope proteins outside the cell membrane, preventing water loss and playing essential roles for epidermis to function as barrier^29^. The upregulation of all these differentiation markers at the transcript and protein levels, along with increase in lipid synthesis, verifies that the mechanical stress coupled with air-exposure accelerated HaCaT differentiation.

## Methods

### Fabrication of the culture device

As shown in the schematic design (Fig. 1), the device was fabricated with an external diameter of 28 mm, internal diameter of 22 mm, and height of 17 mm. After mixing polydimethylsiloxane (PDMS, Sewang Hitech, South Korea) and curing agent (Sewang Hitech, South Korea) at a ratio of 10:1, it was poured into a cast and air bubbles were eliminated using a vacuum pump for 30 min. The cured device was then attached onto a 6-well plate with PDMS bonding and connected to a 14G needle to open the pore under the 6-well plate. The lateral holes of the membrane were punched using a 0.1 mm PDMS punch. Devices were sterilized with 70% alcohol. To prepare the nanofiber, 1.5% (w/v) PCL (Sigma-Aldrich, MO, USA) was dissolved in a mixed solution of 7 ml tetrahydrofuran (Junsei, Japan) and 3 ml N,N-dimethylformamide (Junsei, Japan) and stirred for 24 h. After stirring, 45 μg/ml of C-phycocyanin was added to confer several positive functions (anti-inflammatory, ROS scavenging, etc.) to the cell culture based on our previous study^21^. The distance of the needle tip to the collector was 15 cm, and the solution was electrospun at 1 ml/h and 15 kV. The nanofiber (PCL-NF) was attached to the membrane using PDMS.

### Cell culture and HaCaT differentiation

HaCaT and human dermal fibroblasts (HDF, Thermos Fisher Scientific, MA, USA) were cultured in the culture media (DMEM-high glucose culture media (Thermos Fisher Scientific, MA, USA) supplemented with 10% fetal bovine serum (FBS, Thermos Fisher Scientific, MA, USA) and 1% antibiotic-antimycotic (Thermos Fisher Scientific, MA, USA)), in an incubator with 37 °C, 95% relative humidity and 5% CO_2_. It was hardened by collagen containing 10x EMEM (Lonza, NJ, USA), 200 mM L-glutamine (Thermos Fisher Scientific, MA, USA), FBS, 7.5% sodium bicarbonate (Thermos Fisher Scientific, MA, USA) and bovine collagen type I (Advanced Biomatrix, USA) on the nanofiber layer. A mixture of 4 × 10^5^ cells/ml HDF (passage 4–8) and the aforementioned collagen solution was solidified onto the nanofiber layer for 1 h and then incubated for 4 days in culture media. An epidermal layer was prepared by seeding it with 5 × 10^6^ cells/ml HaCaT, which was incubated in the DMEM supplemented with Ham’s F12 medium (Thermos Fisher Scientific, MA, USA), 200 mM L-glutamine, hydrocortisone (Sigma-Aldrich, MO, USA), 0.05 M O-phosphorylethanolamine (Sigma-Aldrich, MO, USA), 90 mM Adenine (Sigma-Aldrich, MO, USA), 2 nM progesterone (Sigma-Aldrich, MO, USA), 1 M CaCl_2_ (Sigma-Aldrich, MO, USA), 10 nM triiodothyronine (Sigma-Aldrich, MO, USA), and newborn calf serum (Thermos Fisher Scientific, MA, USA). Culture media was changed every two days. After being cultured for 4 days, HaCaT were subjected to air exposure and mechanical stress with intermittent stretching and non-stretching for 14 days. To provide a consistent stimulus to the culture system until harvest, the feed flow rate into the lower chamber was controlled by a programmed strategy using the Arduino-assisted pump. In addition, the system was programmed to take the intermittent stimuli every 30 min because excessive stimuli would adversely affect cellular proliferation and survival^17^. The stretch is precisely applied for 1 min in a 30 min cycle. Cells undergo a radial stretch for 1 min followed by no stretch for 29 min in the cycle. These intermittent stretches are cyclically given for 14 days until cell harvesting.

### Calculation of radial strain changes by membrane extension

Radial strain of membrane indicated the stress level exposed to artificial tissue exactly. The extended membrane was taken in horizontal direction by CCD camera, and then, the length of extended surfaces in images was measured with the help of image J. The radial strain was calculated by the below equation (1).


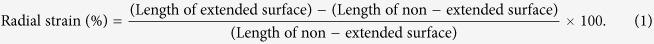


### Fabrication of ceramide nanoparticles

Ceramide/PLGA nanoparticles were fabricated following the previous research^30^: 25 mg poly lactic-co-glycolic acid (PLGA, Sigma-Aldrich, MO, USA) and 10 mg ceramide (Doosan, South Korea) were dissolved in 0.5 ml chloroform (Sigma-Aldrich, MO, USA), then mixed with 10 ml 2.5% (w/v) polyvinyl alcohol (Junsei, Japan). The resultant sample was subjected to sonication (Sonictopia, South Korea) for 30 s, after which the chloroform was removed by stirring at 4 °C for 24 h. Next, 0.5 ml of 10 mg/ml chitosan (Sigma-Aldrich, MO, USA) dissolved in 1% acetic acid (Daejung, South Korea) was added to each sample and allowed to react for 2 h to develop a chitosan coating on the nanoparticle.

### Evaluation of morphological confirmation of keratinocytes

The 3D skins were freeze-dried, after which the keratinized cell surface was observed by scanning electron microscopy (S-4200, Hitachi, Japan). FITC-labeled nanoparticles were treated on 3D skin to observe the side view of the epidermal layer. Briefly, 1 mg/ml FITC solution was prepared and mixed with FITC (Sigma Aldrich, USA) and 200 mM sodium bicarbonate (Thermos Fisher Scientific, USA). After centrifuging 1 ml of chitosan-coated ceramide nanoparticle for 30 min at 4 °C, the supernatant was removed and the collected nanoparticles were resuspended in 1 ml FITC solution, then reacted for 1 h at RT. FITC-labeled nanoparticles were collected by centrifugation for 30 min at 4 °C, after which they were washed with distilled water. Following another round of centrifugation, the resuspended nanoparticles in PBS were dropped on the prepared 3D skin for 1 h at 37 °C. Finally, the 3D skin was washed with PBS and sectioned into OCT blocks. The epidermis morphology was then observed by confocal microscopy.

### Quantification of nucleated cells of 3D skin

3D skin was cultured for 14 days to confirm differences in confluency and number of cells, depending on stretch. The harvested tissues were incubated with 2.5 mg/ml dispase in DMEM at 37 °C for 4 h. After being separated from the 3D skin, epidermis was treated with 0.25% EDTA/trypsin overnight at 4 °C. Next day, trypsin including Hoechst was reacted to stain nucleus of cells at 37 °C for 20 min and neutralized with 10% FBS in DMEM. Single cells were prepared by pipetting and then harvesting by centrifugation at 1000 rpm for 3 min. The pellet was added to 1 ml DMEM for resuspension. Finally, nucleated cells were counted using a hemocytometer.

### qRT-PCR for measuring differentiation markers for HaCaT

qRT-PCR was conducted to examine the expression level of differentiation markers of 3D cultured HaCaT on the device. Primers of keratin 1, filaggrin, involucrin, and loricrin were prepared as keratinocyte differentiation markers and GAPDH was used as a housekeeping gene (Table 1). Total mRNAs were collected using an RNeasy MiNi kit plus (Qiagen, Germany). The mRNA was utilized to synthesize cDNA using a QuantiTecT Reverse Transcription kit (Qiagen, Germany). The cDNA was then subjected to qRT-PCR using a Rotor-Gene Q PCR (Qiagen, Germany).

### Confirmation of protein level by immunofluorescence assay and lipid contents of stratum corneum

The 3D skins were fixed in 4% paraformaldehyde dehydrate (Sigma-Aldrich, MO, USA), then submerged in 30% sucrose (USB Corporation, OH, USA) solution for 1 day, followed by in 50% solution for another day. Next, the dehydrated 3D skins were frozen using OCT compound (Sakura, Netherlands) at −80 °C. The sliced OCT samples, with a thickness of 40 μm, were dried for 1 day at −20 °C. Cryosectioned samples were washed 2 times with iced phosphate-buffered saline (PBS), treated with PBST (PBS supplemented with 0.05% Tween 20 (USB Corporation, OH, USA) and 1% bovine serum albumin (Sigma-Aldrich, MO, USA)) for 30 min at room temperature (RT), then incubated with diluted filaggrin (Abcam, UK) and loricrin antibody (Abcam, UK) at RT for 1 h. After washing 3 times with iced PBS, samples were treated with donkey polyclonal secondary antibody (Abcam, UK) diluted with PBST for 1 h in the dark at RT. Confocal laser scanning microscopy (LSM 510 meta, Carl Zeiss, Germany) was then used to confirm the differentiation markers. Additionally, Nile red staining was used to confirm the lipid content in the stratum corneum. Briefly, samples were fixed with 4% paraformaldehyde overnight. The 3D skins were treated with a mixture of PBS and 2 μg/ml Nile red dye (Sigma-Aldrich, MO, USA) at a 3:1 ratio. The samples were incubated for 10 min at 40 °C in the dark and observed using confocal laser scanning microscopy. The quantified data of fluorescent intensity was measured with image J program.

### Statistical analysis

All experiments were conducted 7 times, after which *t*-tests were performed to confirm the relationship based on the average value ± the standard deviation (*p < 0.05, **p < 0.01, ***p < 0.001).

## Additional Information

**How to cite this article**: Jung, M. H. *et al*. Co-stimulation of HaCaT keratinization with mechanical stress and air-exposure using a novel 3D culture device. *Sci. Rep.* **6**, 33889; doi: 10.1038/srep33889 (2016).

## Supplementary Material

Supplementary Information

Supplementary Video S1

## Figures and Tables

**Table 1:** Oligonucleotide sequences used in qRT-PCR.

Gene	Forward (5′→3′)	Reverse (5′→3′)
GAPDH	ACCCACTCCTCCACCTTTG	CTCTTGTGCTCTTGCTGGG
Keratin 1	TGACAAGGTGAGGTTCCTGG	GTTGGTCCACTCTCCTTCGG
Filaggrin	GCTTTCTGTGCTTGTGTCCT	TGATGTCTTCATGGATCACT
Involucrin	CTGCCTCAGCCTTACTGTGA	GGAGGAACAGTCTTGAGGAG
Loricrin	GCAAACCTCGGGTAGCATCA	GCCGTCCAAATAGATCCCCC
