# Supplementary material for: Co-stimulation of HaCaT keratinization with mechanical stress and air-exposure using a novel 3D culture device
Source: Sci Rep. 2016 Sep 27;6:33889. doi: 10.1038/srep33889 (PMC5037429; doi:10.1038/srep33889)
Supplement: Supplementary Information [file srep33889-s1.pdf]

Co-stimulation of HaCaT keratinization with mechanical stress and air-exposure using a novel 3D culture device

Moon Hee Jung[1], Sang-Myung Jung[1], Hwa Sung Shin[1,*]

[1]Department of Biological Engineering, Inha University, Incheon, 402-751, Korea

*Corresponding author.

Hwa Sung Shin, hsshin@inha.ac.kr, Tel: 82-32-860-9221, Fax: 82-32-872-4046

This file includes:

1 video and 3 figures which are supporting our research.

Supplementary Video S1 Demonstration of the operation of the all-in-one device.

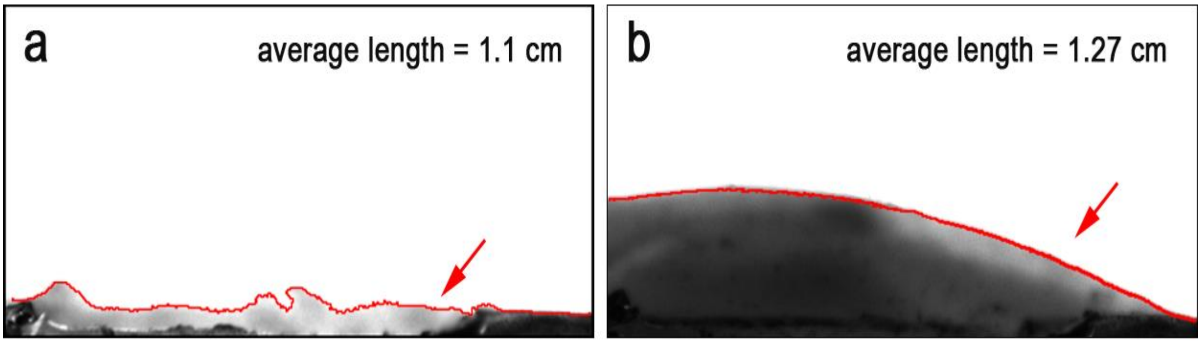

Supplementary Figure S1 Images of non-extended (a) and extended membrane (b) of device. For extended membrane, 1 ml of water was input into lower chamber. The images were taken by CCD camera at horizontal direction to observe fully extended line of membrane. Radial strain was calculated after directly measuring and comparing lengths of both membranes.

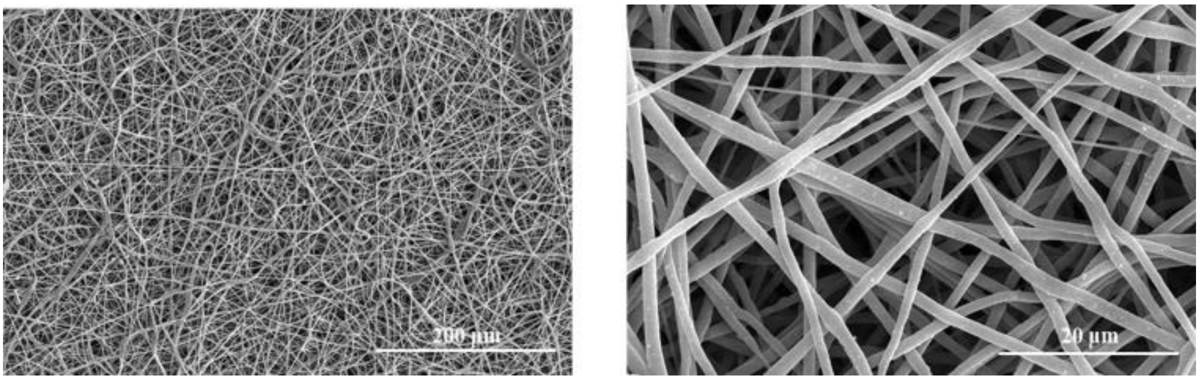

Supplementary Figure S2 Nanofiber for stabilizing collagen-fibroblast matrix. PCL-NF prevented contraction of collagen since it acted as a scaffold to fix the matrix in place. PCL-NF was incorporated with C-phycocyanin inducing several positive functions such as anti-inflammation and ROD scavenging. The concentration of C-Pc was referred to a previous study. Bar = 200 μm, 20 μm

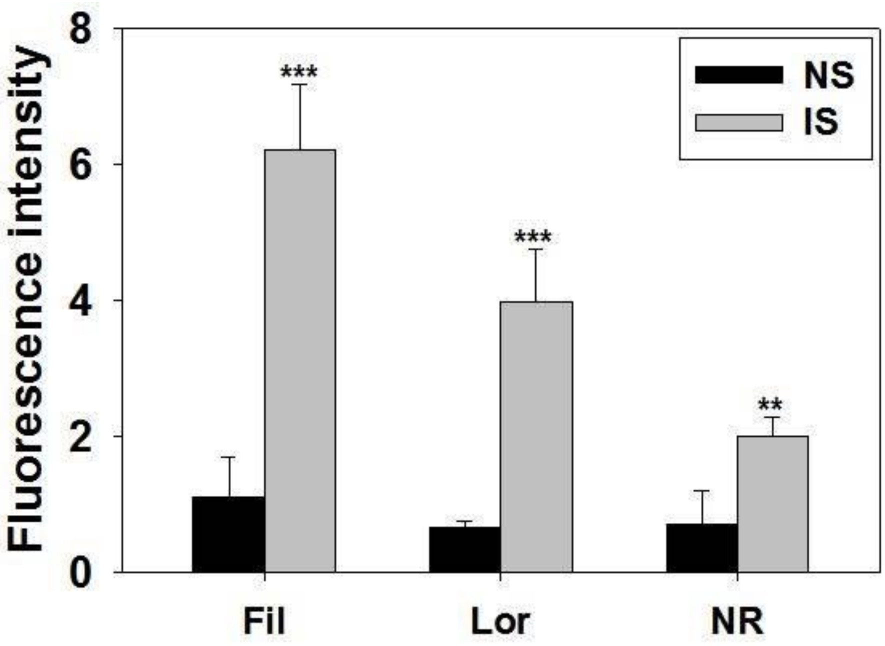

Supplementary Figure S3 The fluorescence intensity was quantified with the help of Image J program. Filaggrin and loricrin in IS were higher than NS. Lipid contents of stratum corneum increased in IS, relative to NS (*p < 0.05, **p < 0.01, ***p<0.001).
